# Amyloid-*β* clearance: an astrocytic perspective

**DOI:** 10.3389/fnins.2026.1812081

**Published:** 2026-05-07

**Authors:** Jiayi Li, Zhongyue Lv, Xiao Chen, Fan Fu, Kaixia Yang, Guogang Lai, Hao Wu, Guomin Xie

**Affiliations:** 1 Department of Neurology, The Affiliated Lihuili Hospital of Ningbo University, Ningbo University, Ningbo, Zhejiang, China; 2 Ningbo Institute of Innovation for Combined Medicine and Engineering (NIIME), The Affiliated Lihuili Hospital of Ningbo University, Ningbo University, Ningbo, Zhejiang, China

**Keywords:** Alzheimer’s disease, astrocyte, A*β* clearance, blood–brain barrier, glymphatic pathway, neuroinflammation

## Abstract

Alzheimer’s disease (AD) represents a major global public health challenge in the 21st century, recognized as the neurodegenerative disorder with the highest mortality rate and socioeconomic burden. The core pathological feature of AD is an imbalance in A*β* production and clearance, leading to conformational changes and pathological aggregation of A*β* peptides. This imbalance triggers neurodegenerative cascades 20–30 years before clinical symptoms appear. Therapeutic approaches targeting A*β* production, including *β*-secretase and *γ*-secretase inhibitors, have thus far shown limited clinical benefit in late-stage trials and have been further constrained by safety and tolerability concerns. As a result, early interventions aimed at enhancing A*β* clearance have attracted increasing attention. While microglia-mediated phagocytosis of A*β* has been extensively studied, the multifaceted roles of astrocytes in this process remain underexplored. This review synthesizes recent findings to elucidate the molecular mechanisms of astrocyte-mediated A*β* clearance, focusing on endocytic uptake and intracellular degradation, maintenance of the blood–brain barrier, and aquaporin-4 (AQP4)-dependent glymphatic drainage. Additionally, this review dissects key regulatory nodes, including the dynamic modulation of A*β* clearance capacity through astrocyte phenotypic transitions and functional decline associated with pathology. These insights offer a theoretical foundation and translational perspective for the development of astrocyte-targeted interventions in early-stage AD.

## 1 Alzheimer’s disease and A*β* pathology

Alzheimer’s disease (AD) is a progressive neurodegenerative disorder marked by severe cognitive decline. A key driver of its pathogenesis is the dysregulation of amyloid-*β* (A*β*) homeostasis, which involves an imbalance between A*β* production and clearance (Hardy and Selkoe, 2002; Mawuenyega et al., 2010). Under normal conditions, amyloid precursor protein (APP) processing predominantly follows the non-amyloidogenic pathway, where cleavage by *α*-secretase within the A*β* sequence prevents the formation of full-length A*β* peptides, producing soluble, monomeric fragments with neuroprotective properties (Esler and Wolfe, 2001; Giuffrida et al., 2009). In contrast, in the pathological state, APP undergoes sequential cleavage by *β*- and *γ*-secretases (the amyloidogenic pathway) (Esler and Wolfe, 2001). This process generates A*β* peptides of different lengths, predominantly A*β*40 and A*β*42, which can misfold and assemble into multiple aggregation states, including soluble oligomers, protofibrils, and insoluble fibrils with increasing *β*-sheet-rich structure (Esler and Wolfe, 2001; Hardy and Selkoe, 2002).

Among these species, A*β*42 is considered particularly pathogenic because its greater hydrophobicity and stronger aggregation propensity favor the formation of neurotoxic soluble oligomers and fibrillar deposits, whereas A*β*40, although more abundant, is more often linked to vascular amyloid accumulation (Esler and Wolfe, 2001; Hardy and Selkoe, 2002). Although fibrillar A*β* contributes prominently to senile plaque formation, soluble A*β* oligomers are now widely regarded as the principal neurotoxic species in AD. These oligomers initiate a pathological cascade that includes Tau hyperphosphorylation, NFT formation, glial activation, chronic neuroinflammation, synaptic dysfunction, and neuronal loss (Hardy and Selkoe, 2002; Linnerbauer et al., 2020; Scheltens et al., 2021). Collectively, these pathological events ultimately manifest clinically as progressive cognitive decline and dementia (Hardy and Selkoe, 2002; Villemagne et al., 2013).

Given the strong correlation between A*β* burden and cognitive decline, restoring A*β* homeostasis has become a central therapeutic target (Villemagne et al., 2013). However, the mechanisms underlying A*β* accumulation differ between familial and sporadic AD. Familial AD is primarily associated with Amyloid Precursor Protein (APP), Presenilin 1 (PSEN1), or Presenilin 2 (PSEN2) mutations that increase A*β* production or promote the generation of more aggregation-prone species, particularly A*β*42 (Lanoiselée et al., 2017). In contrast, growing evidence suggests that impaired A*β* clearance, rather than excessive production, may be the primary pathogenic factor in sporadic AD, which accounts for over 90% of AD cases, while impaired clearance is also observed in familial AD (Bateman et al., 2006; Mawuenyega et al., 2010; Lanoiselée et al., 2017). Because *β*- and *γ*-secretases are key enzymes in A*β* generation, early therapeutic strategies focused on inhibiting these enzymes to reduce A*β* production. However, clinical trials of *β*- and γ-secretase inhibitors such as semagacestat and verubecestat showed limited efficacy and were further constrained by adverse effects and tolerability concerns (Doody et al., 2013; Egan et al., 2019). At the same time, FDA-approved anti-A*β* monoclonal antibodies such as lecanemab and donanemab have shown a degree of clinical benefit in early symptomatic AD, potentially through activating microglia to enhance the clearance of aggregated A*β*, as demonstrated in the phase 3 CLARITY-AD and TRAILBLAZER-ALZ 2 trials (Sims et al., 2023; van Dyck et al., 2023). These studies mark a paradigm shift in AD treatment strategy—from suppressing A*β* synthesis to restoring its clearance capacity.

Cerebral A*β* clearance operates through three principal mechanisms: (1) cellular uptake and enzymatic degradation, (2) receptor-mediated transport across the blood–brain barrier (BBB), and (3) convective exchange and drainage via the glymphatic system (Tarasoff-Conway et al., 2015). Although microglia have traditionally been emphasized as the principal phagocytes involved in A*β* uptake, astrocytes are uniquely positioned to regulate A*β* homeostasis across all three pathways through their roles in cell degradation, BBB support, and AQP4-dependent glymphatic function (Allen and Lyons, 2018; Brandebura et al., 2023). Notably, experimental disruption of astrocyte-associated clearance pathways has been shown to impair brain A*β* removal and exacerbate amyloid pathology, including in astrocyte-specific LRP1-deficient APP/PS1 mice and in models with ablated reactive astrocytes (Liu C. -C. et al., 2017; Katsouri et al., 2020). This broader systems-level involvement provides the rationale for focusing the present review on astrocyte-mediated A*β* clearance.

## 2 Heterogeneity of reactive astrogliosis and A*β* clearance

Astrocytes, derived from neuroepithelial radial glial cells, represent the most abundant glial population in the mammalian brain, comprising approximately 30% of all central nervous system (CNS) cells (Allen and Lyons, 2018). Their regulatory functions are extensive, including: providing energetic support to neurons and other glial cells via the lactate shuttle mechanism, in which astrocytes convert glucose to lactate and shuttle it to neurons as an energy substrate (Magistretti and Allaman, 2018); maintaining extracellular ion and pH homeostasis (Theparambil et al., 2020), and regulating excitatory–inhibitory (E/I) balance via neurotransmitter uptake (e.g., glutamate) (Sidoryk-Węgrzynowicz et al., 2024); modulating synaptic plasticity, essential for learning and memory, *via* gliotransmitters (e.g., adenosine, D-serine) (Arizono et al., 2020); participating in the neurovascular unit (NVU) and glymphatic system, where they regulate cerebral blood flow (CBF) and facilitate the circulation and clearance of metabolic waste (Obermeier et al., 2013; Lohela et al., 2022); and coordinating neuroinflammatory responses and clearing pathological proteins under pathological conditions (Linnerbauer et al., 2020).

With the development of single-nucleus RNA sequencing, researchers have revealed that astrocytes exhibit complex, multidimensional heterogeneity. This includes spatial heterogeneity, in which astrocytic states depend on the brain region they occupy and their physical proximity to pathological lesions, such as A*β* amyloid plaques in AD (Chen et al., 2020); temporal heterogeneity, whereby astrocytes may exert protective effects during the early stages of disease, but progressively lose their homeostatic functions and may even acquire neurotoxic properties as the disease advances (Serrano-Pozo et al., 2024); and molecular heterogeneity, characterized by distinct gene expression profiles and activation of different signaling pathways (Chen et al., 2020; Serrano-Pozo et al., 2024). Accordingly, Escartin et al. proposed a consensus term, reactive astrocytes, to describe astrocytes that undergo molecular, morphological, and functional remodeling under pathological conditions (Escartin et al., 2021). Moreover, their different reactive states should be defined according to molecular lineage, functional phenotype, and specific effects on pathology, going beyond the traditional binary classification of astrocytes into A1 (neurotoxic) and A2 (neuroprotective) states (Escartin et al., 2021). Subsequent work refined this framework in AD by showing that astrocytes undergo dynamic state transitions. Habib et al. identified disease-associated astrocytes in AD models, marked by transcriptional changes including *APOE and Clu* (Habib et al., 2020), whereas Serrano-Pozo et al. further demonstrated in human AD brains a continuum from homeostatic to intermediate and reactive astrocyte states across the spatiotemporal progression of pathology (Serrano-Pozo et al., 2024).

The dynamic process of reactive astrogliosis directly influences A*β* clearance efficiency, reflecting the complex balance between adaptation and maladaptation to A*β* toxicity (Habib et al., 2020; Escartin et al., 2021; Brandebura et al., 2023). Studies demonstrate that in APP/ArcSwe transgenic mice, with disease progression, reactive astrocytes develop lysosomal dysfunction and fail to degrade engulfed A*β* aggregates, leading to aberrant endosomal enlargement and release of neurotoxic extracellular vesicles that propagate pathology to neighboring neurons (Söllvander et al., 2016). Concurrently, studies in APP/PS1 mice have shown that reactive astrogliosis leads to AQP4 depolarization—the mislocalization of AQP4 away from perivascular endfeet—thereby impairing convective exchange and drainage via the glymphatic system (Xu et al., 2015). Furthermore, reactive astrogliosis generates neuroinflammatory cascades that disrupt the BBB integrity, suppressing A*β* clearance (Yue and Hoi, 2023). In this context, reactive astrocytes act not merely as responders to pathology but as active propagators of it, while the worsening amyloid and inflammatory milieu further reinforces astrocyte dysfunction, creating a self-perpetuating feed-forward cycle (Arranz and Strooper, 2019; Linnerbauer et al., 2020; Escartin et al., 2021; Serrano-Pozo et al., 2024).

Thus, AD is not solely a neuronal disorder, but also reflects functional dysregulation of astrocyte-mediated homeostatic regulation (Brandebura et al., 2023). Astrocytic dysfunction is no longer viewed as a secondary consequence of AD pathology but may constitute a core driver of disease pathogenesis (Allen and Lyons, 2018). As astrocytes participate in A*β* clearance through three complementary pathways—(1) intracellular degradation and extracellular enzymatic breakdown, (2) maintenance of BBB integrity and receptor-mediated transcytosis, and (3) facilitation of glymphatic clearance—their functional integrity is essential for effective A*β* clearance and disease modification (Arranz and Strooper, 2019; Brandebura et al., 2023). In the following sections, we summarize these astrocyte-mediated A*β* clearance pathways and their pathological dysregulation.

## 3 Degradation clearance

### 3.1 Cellular internalization of A*β*

Astrocytes utilize various mechanisms to internalize A*β*, with the selection of pathways largely depending on the aggregation state of the peptide. These mechanisms include phagocytosis, macropinocytosis, and endocytosis, with clathrin-mediated endocytosis (CME) playing a central role in the clearance of soluble A*β* (sA*β*). CME is a highly specific, regulated process essential for the homeostatic removal of soluble monomers and oligomers (Table 1).

**Table 1:** Comparison of three A*β* internalization mechanisms in astrocytes.

Feature	Clathrin-mediated endocytosis (CME)	Macropinocytosis	Phagocytosis
Main A*β* substrate	A*β* monomers; possibly small soluble A*β* oligomers (Tachibana et al., 2019; Martens et al., 2022)	monomeric A*β*42 and oligomeric/protofibrillar A*β*42 (Wesén et al., 2017; Nazere et al., 2022)	A*β* oligomers (Zhang et al., 2014) and larger A*β*-associated particles, including plaque-associated dystrophic neurites/A*β*-containing neuronal debris (Chung et al., 2013; Sanchez-Mico et al., 2021)
Core mechanism	Receptor-mediated internalization through clathrin-coated pits (Wesén et al., 2017).	Actin-driven membrane ruffling and non-selective fluid-phase uptake (Hewlett et al., 1994).	Actin-dependent engulfment of large particles via receptor-mediated phagocytic wrapping (Cramer et al., 2012; Chung et al., 2013).
Vesicle characteristics	Small vesicles (generally 100–150 nm) (Heymann et al., 2013). Coat proteins: Clathrin, AP-2 (Bres and Faissner, 2019)	Large “macropinosomes” (generally > 0.2 μm) (Hewlett et al., 1994; Swanson and Watts, 1995). Coat proteins: None. Relies on actin/membrane remodeling (Swanson and Watts, 1995; Kay et al., 2024).	Large “phagosomes” (generally > 0.5 μm); size depends on particle (Chung et al., 2013; Konishi et al., 2022). Coat proteins: None. Relies on actin cytoskeleton (Konishi et al., 2022).
Key receptors	LRP1 (Bres and Faissner, 2019). LDLR (Kim et al., 2009; Basak et al., 2012). LRP2 (Megalin) (Takano-Kawabe et al., 2023).	Non-receptor-mediated endocytosis (Hewlett et al., 1994; Kay et al., 2024).	MEGF10 and MERTK (Chung et al., 2013; Sanchez-Mico et al., 2021). Scavenger Receptors (Alarcón et al., 2005; Zhang et al., 2014).
Consensus role in astrocytes	Homeostatic clearance, maintaining brain A*β* balance	High-capacity, emergency clearance activated under high A*β* concentrations; may induce astrocytic stress	Clearance of large pathological structures, protecting the neuronal environment

#### 3.1.1 Phagocytosis

Phagocytosis is a receptor-mediated process specialized for the uptake of large solid particles, such as amyloid aggregates, pathogens, and cellular debris (Chung et al., 2013). In the context of AD, reactive astrocytes undergo cytoskeletal remodeling and extend pseudopodia, enabling them to engulf plaque-associated dystrophic neurites and A*β*-containing neuronal debris, thereby contributing to indirect A*β* clearance (Chung et al., 2013; Sanchez-Mico et al., 2021). Current evidence primarily identifies MEGF10 and MERTK as the core astrocytic phagocytic receptors mediating the engulfment of synapses and plaque-associated dystrophic neurites/debris (Chung et al., 2013), whereas scavenger receptors, ApoE, and complement are thought to play modulatory roles (Alarcón et al., 2005; Chung et al., 2016); however, direct evidence for their involvement in the uptake of dense-core A*β* plaques remains limited (Chung et al., 2013; Zhang et al., 2023). Nevertheless, rather than migrating to plaques, astrocytes primarily respond through local phenotypic remodeling, likely limiting their access to dense A*β* cores (Galea et al., 2015). Moreover, compared with professional phagocytes such as microglia, astrocytes exhibit relatively limited degradative efficiency toward internalized aggregated A*β*, creating a “rapid uptake, slow digestion” imbalance that promotes pathological intracellular A*β* accumulation (Chung et al., 2013; Söllvander et al., 2016; Zhang et al., 2023).

#### 3.1.2 Macropinocytosis

Macropinocytosis is a non-selective form of fluid-phase endocytosis characterized by actin-dependent membrane ruffling and the engulfment of large volumes of extracellular fluid (Kay et al., 2024). In aU87-MG human glioblastoma cells, both soluble monomeric and oligomeric/protofibrillar A*β*42 have been reported to enter cells through fluid-phase macropinocytosis, and this process is also dependent on dynamin2 (Li et al., 2014). Additional studies in neuronal cell models further suggest that oligomeric A*β* uptake may involve HSPG- and lipid raft-dependent mechanisms and Rac1-associated macropinocytic signaling (Wesén et al., 2017; Nazere et al., 2022). However, these mechanisms require further validation in astrocytes.

#### 3.1.3 Clathrin-mediated endocytosis

CME is a highly efficient, receptor-mediated pathway for astrocytic clearance of sA*β*, particularly the neurotoxic oligomeric species (Lee et al., 2015; Domínguez-Prieto et al., 2018). Pharmacological evidence supports this mechanism: actin polymerization inhibitors like Cytochalasin B do not block sA*β* internalization, indicating that the primary uptake route is independent of actin-dependent classical phagocytosis. In contrast, CME inhibitors, including chlorpromazine (CP), significantly reduce astrocytic A*β* internalization, particularly for neurotoxic soluble A*β*42 oligomers (Lee et al., 2015). This selective clearance likely results from the size constraints of canonical CME vesicles, which enable the pathway to effectively limit the accumulation of highly neurotoxic A*β* forms during pre-plaque pathological stages (Heymann et al., 2013).

**Figure 1:**
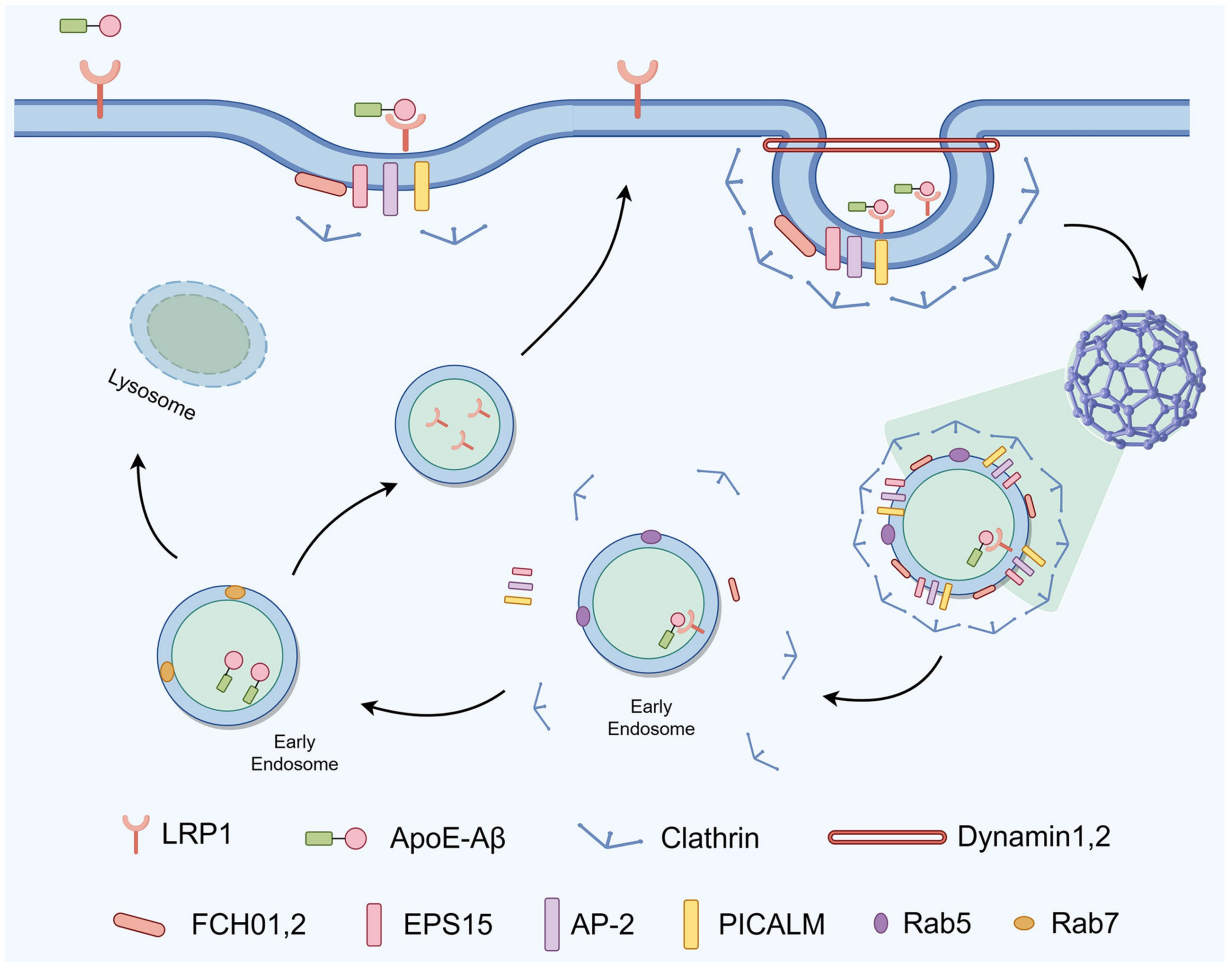
Schematic representation of CME-dependent A*β* internalization in astrocytes.

The CME process begins with the specific recognition of extracellular complexes, such as those formed by ApoE and A*β*, by cell surface receptors like LRP1 (Figure 1). During this initiation phase, early nucleator proteins FCHO1/2 bind to the inner leaflet of the plasma membrane, recruiting EPS15 (Kaksonen and Roux, 2018). This complex, in turn, facilitates the aggregation of adaptor proteins AP-2 and PICALM on the inner membrane, leading to the recruitment of clathrin and the formation of a “clathrin-coated pit” (Kaksonen and Roux, 2018). As the process continues, BIN1 senses membrane curvature and aids in membrane bending. Dynamin then hydrolyzes GTP to sever the vesicle neck, ultimately forming an intracellular vesicle that encapsulates A*β* (Kaksonen and Roux, 2018). Endosomes mature through a Rab5-to-Rab7 conversion, releasing the corresponding receptors, and ultimately fuse with lysosomes to clear internalized A*β*.

##### 3.1.3.1 Core receptors in astrocytic CME

CME relies on the coordinated action of specific receptors and molecular chaperones, particularly low-density lipoprotein receptor-related protein 1 (LRP1) and the molecular chaperone ApoE. LRP1 is the core receptor in astrocytes that regulates A*β* metabolism (Bres and Faissner, 2019; Li et al., 2025), capable of binding A*β* peptides directly and can also facilitate A*β* binding through molecular partners such as apolipoprotein E (ApoE), Clusterin (ApoJ), and α2-macroglobulin (Bres and Faissner, 2019). Beyond mediating endocytosis, LRP1 deficiency significantly downregulates the expression of A*β*-degrading enzymes, including MMP-2, MMP-9, and IDE, resulting in a “double failure” of both A*β* uptake and degradation (Liu C. -C. et al., 2017). Multiple studies have shown that astrocyte-specific LRP1 knockout exacerbates amyloid plaque deposition and neurodegeneration (Liu C. -C. et al., 2017; Tachibana et al., 2019; Zang et al., 2021).

In addition to LRP1, several other receptors are involved in astrocytic A*β* internalization. Low-density lipoprotein receptor (LDLR) efficiently mediates the uptake of ApoE–A*β* complexes, thereby promoting their trafficking to lysosomes (Kim et al., 2009; Basak et al., 2012). This role has been validated in AD mouse models, in which LDLR overexpression was shown to reduce A*β* burden and alleviate neuroinflammation (Kim et al., 2009). In addition, LRP2 (megalin) has also been implicated in the endocytic clearance of specific A*β*-associated complexes in cultured astrocytes derived from rat embryonic cortex (Takano-Kawabe et al., 2023).

##### 3.1.3.2 ApoE as the principal chaperone in astrocytic CME

ApoE, the primary molecular chaperone in the CNS, is predominantly produced by astrocytes (Zhao et al., 2018; Martens et al., 2022; Golden et al., 2024). Its lipidation via the ATP-binding cassette transporter A1 (ABCA1) transporter is essential for forming functional HDL-like lipoprotein particles, the structural basis for mediating A*β* clearance (Kanekiyo et al., 2014; Zhao et al., 2018; Golden et al., 2024). In the context of AD, the human APOE gene encodes three major isoforms: protective APOE2/3 and risk-associated APOE4, with ε3 being the most common allele (Kanekiyo et al., 2014; Zhao et al., 2018; Golden et al., 2024). Normal variants undergo full lipidation to bind LRP1 with high affinity, mediating rapid endosomal-lysosomal degradation of ApoE–A*β* complexes while maintaining A*β* solubility and preventing aggregation into neurotoxic oligomers (Kumar et al., 2016). Additionally, studies have shown that ApoE2 and ApoE3 can promote proteolytic degradation of A*β* by activating neprilysin (NEP) and insulin-degrading enzyme (IDE) (Jiang et al., 2008).

In contrast, ApoE4 promotes ADP-ribosylation factor 6 (ARF6) overexpression, which retains ABCA1 in late endosomes instead of allowing its recycling to the plasma membrane (Lee et al., 2022; Martens et al., 2022). As a result, the secreted ApoE is poorly lipidated and conformationally defective. This reduces its binding affinity for A*β* and shifts A*β*-containing complexes from the rapid LRP1 pathway to VLDLR, which internalizes cargo more slowly and tends to favor recycling over degradation (Deane et al., 2008). Together, these changes impair A*β* clearance. Additionally, studies in human APOE ε4 astrocytes have shown that downregulation of NHE6 causes endosomal hyperacidification, resulting in intracellular retention of LRP1 and markedly reduced cell surface expression, thereby inhibiting A*β* binding and endocytosis (Prasad and Rao, 2018a,b). This process may be mediated by HDAC4 nuclear translocation and may further perturb downstream endosomal–lysosomal trafficking (Prasad and Rao, 2018a).

##### 3.1.3.3 ApoE-dependent astrocyte–microglia crosstalk in A*β* clearance

Beyond affecting astrocytic A*β* uptake itself, astrocyte-derived ApoE also regulates microglia-mediated A*β* clearance. Studies have shown that ApoE2 and ApoE3 bind to TREM2 on microglia, thereby promoting their transition toward a disease-associated microglia (DAM) phenotype and enhancing microglial phagocytic and lysosomal activity, both of which are required for effective A*β* engulfment and plaque compaction (Krasemann et al., 2017; Fitz et al., 2021). In contrast, ApoE4 disrupts this intercellular coordination through multiple converging mechanisms: it impairs TREM2 signaling and downregulates the microglial chemotactic receptor P2RY12, thereby reducing microglial recruitment toward A*β* deposits and weakening plaque containment (Parhizkar et al., 2019; Fitz et al., 2021). Meanwhile, ApoE4 upregulates ACSL1, an enzyme that promotes fatty-acid activation and lipid droplet biogenesis, contributing to lysosomal dysfunction (Victor et al., 2022). As a result, ApoE4 microglia exhibit a dystrophic morphology, characterized by swollen cell bodies, together with severely impaired phagocytic capacity (Xiao et al., 2014; Parhizkar et al., 2019).

These two glial cell types are also embedded in a broader network of bidirectional communication, including extensive crosstalk through complement cascades (e.g., C1q and C3), inflammatory cytokines, and metabolite shuttling, which together shape the neuroinflammatory microenvironment and synaptic integrity (Liddelow et al., 2017; Linnerbauer et al., 2020). Dysregulation of this intercellular dialog, particularly in the ApoE4 context, can amplify pro-inflammatory signaling and exacerbate neuritic dystrophy independently of direct A*β* toxicity (Liddelow et al., 2017; Linnerbauer et al., 2020; Golden et al., 2024). Although the detailed molecular mechanisms of astrocyte–microglia metabolic coupling are beyond the scope of this review, it should be emphasized that astrocytes are the principal source of ApoE, whereas microglia are major executors of ApoE-dependent A*β* clearance. Therefore, ApoE constitutes a critical node in astrocytic regulation of A*β* homeostasis, and its functional integrity is essential for maintaining the overall efficiency of the A*β* clearance system (Prasad and Rao, 2018a; Martens et al., 2022).

While phagocytosis, macropinocytosis and receptor-mediated endocytosis efficiently internalizes A*β* into astrocytes, this process represents only the initial step of clearance (Li et al., 2014; Söllvander et al., 2016; Tachibana et al., 2019). However, in the absence of a robust intracellular degradation system, excessive A*β* uptake may paradoxically lead to lysosomal overload, which amplifies inflammatory signaling, triggers apoptosis, and potentially accelerates the spread of A*β* plaques (Söllvander et al., 2016). Therefore, enhancing both astrocytic tolerance and intracellular degradative capacity is essential to ensure the safety and efficacy of A*β* clearance strategies.

### 3.2 Intracellular degradation

Proteostasis is essential for the supportive functions of astrocytes, with the endosomal-lysosomal and autophagy-lysosome pathways playing pivotal roles in A*β* degradation.

#### 3.2.1 The endosome-lysosome pathway

The endosomal–lysosomal system is the principal intracellular pathway by which astrocytes internalize, sort, and degrade exogenous A*β* (Xiao et al., 2014; Söllvander et al., 2016). After entering astrocytes through multiple uptake pathways, A*β* is first delivered to Rab5-positive early endosomes, where it undergoes initial sorting, then matures into Rab7-positive late endosomes, and is ultimately transported to lysosomes for degradation (Rink et al., 2005). Endosomal maturation, pH homeostasis, and receptor recycling together determine the efficiency of A*β* processing. An important upstream pathological feature of AD is the morphological enlargement of early endosomes (EEs) and their subsequent trafficking arrest (Rink et al., 2005; Söllvander et al., 2016). In astrocytes, endosomal hyperacidification caused by NHE6 downregulation in the APOE4 background constitutes a pathological mechanism specific to this cell type, impairing endosomal maturation and downstream fusion with lysosomes while preventing efficient recycling of LRP1 and other receptors back to the cell surface (Prasad and Rao, 2018a,b). In addition, Rab5/Rab7 dysregulation is an important mechanism underlying impaired endosomal maturation in AD and has been identified primarily in neurons: Rab5 hyperactivation in Down syndrome models involves *β*-CTF–mediated signaling potentiated by oxidative stress (Chen et al., 2025), while defective Rab7 maturation in AD neurons has been linked to CCZ1–MON1A complex dysfunction (Cai et al., 2022)—but these findings provide an important reference for understanding astrocytic endosomal maturation defects. Therefore, restoring astrocytic endosomal function, particularly correcting NHE6-mediated pH dysregulation, represents a critical strategy for enhancing A*β* clearance and slowing AD progression.

#### 3.2.2 The autophagy-lysosomal pathway

Macroautophagy, the primary pathway for degrading intracellular macromolecular aggregates and damaged organelles (Ferrari et al., 2024), plays a critical role in clearing various conformations of A*β*. Unlike neurons, astrocytes exhibit a more pronounced plastic autophagic response to A*β* pathology. Transcriptomic analysis of APP/PS1 mouse models reveals that A*β*-induced pathology leads to the upregulation of key autophagy genes such as MAP1LC3B (encoding LC3B) and SQSTM1 (encoding p62) (Kim et al., 2024). Gain- and loss-of-function experiments confirm the protective efficacy of autophagy: overexpression of LC3B in astrocytes significantly reduces A*β* burden and rescues cognitive deficits, while knockdown of MAP1LC3B or SQSTM1 exacerbates plaque deposition and neuronal loss (Kim et al., 2024). Additionally, astrocytes appear to utilize autophagy to activate the urea cycle and putrescine-degradation pathway, thereby detoxifying ammonia generated during A*β* metabolism through its conversion into urea and downstream metabolites (Frame and Cumming, 2022). This may serve as a core line of defense for maintaining cellular homeostasis and mitigating AD pathology (Frame and Cumming, 2022; Kim et al., 2024).

During AD progression, the autophagy–lysosomal pathway undergoes progressive dysfunction from the early stages onward (Kim et al., 2024). The core mechanisms include: oA*β*-induced aberrant activation of mTORC1 signaling, which promotes phosphorylation and cytoplasmic sequestration of transcription factor EB (TFEB), thereby suppressing autophagy-related gene transcription and impairing lysosomal biogenesis (Kim et al., 2024). In parallel, dysfunction of Rab7, the HOPS tethering complex, and SNARE machinery assembly (STX17–SNAP29–VAMP8) disrupts autophagosome–lysosome fusion, resulting in the accumulation of undegraded cargo (Ktistakis, 2016; Zhang et al., 2021). In contrast to the general mechanisms described above, APOE4 specifically suppresses the transcription factor FoxO3a-mediated autophagy and mitophagy programs in astrocytes, concomitant with loss of mitochondrial membrane potential and ATP depletion, whereas this pathway remains relatively intact in neurons and microglia (Sohn et al., 2021). Overall, astrocytes develop marked autophagic dysfunction during AD progression, and restoring autophagic capacity may therefore represent an important therapeutic strategy.

#### 3.2.3 Lysosomes

Lysosomes are the primary sites of intracellular A*β* degradation and the shared endpoint of the endosomal and autophagic pathways (Lo and Zeng, 2023). In AD, however, astrocytes commonly exhibit lysosomal dysfunction, arising from the combined effects of excessive accumulation of poorly degradable A*β*, disrupted lipid/cholesterol trafficking, chronic neuroinflammation and defective acidification (Zeng et al., 2025).

Excessive A*β* uptake itself may impair astrocytic function (Söllvander et al., 2016; Zeng et al., 2025). Although astrocytes can internalize substantial amounts of A*β* protofibrils, they often favor storage over efficient degradation, leading to persistent intralysosomal A*β* accumulation, insoluble aggregate formation, and enlargement of the endolysosomal system (Söllvander et al., 2016). Human studies further suggest that incompletely degraded A*β* may undergo truncation and repackaging within the endolysosomal pathway, generating more degradation-resistant proteoforms such as N-terminally truncated A*β*2-x (Beretta et al., 2024). Notably, A*β*3(pE)-42 is efficiently taken up by astrocytes but remains poorly degradable (Wirth et al., 2024); its accumulation can trigger cytosolic leakage of lysosomal proteases, thereby promoting pro-inflammatory signaling and synapse loss (Wirth et al., 2024). Overall, excessive uptake and incomplete degradation of A*β* disrupt the astrocytic lysosomal system and may facilitate extracellular spreading through exocytosis, thereby contributing to plaque propagation and worsening AD pathology (Söllvander et al., 2016).

In addition, ApoE4-related defects in lipid trafficking may further aggravate lysosomal injury in astrocytes. Studies show that ApoE4 promotes the retention of ABCA1 in late endosomes, thereby impairing its membrane recycling and cholesterol efflux (Lee H. et al., 2023); the resulting cholesterol accumulation further disrupts mitochondrial homeostasis and suppresses oxidative phosphorylation (Lee H. et al., 2023). More recent work has linked cholesterol accumulation, caveolin-1 upregulation, lysosomal retention of ABCA1, mTORC1 activation, senescence-like changes, and neuroinflammation into a continuous pathological axis, highlighting the pathological relevance of the ABCA1–caveolin-1 interaction in astrocytes (Xiao et al., 2014).

Among these mechanisms, lysosomal acidification defects have gained increasing attention in AD (Lo and Zeng, 2023; Zeng et al., 2025). Studies in AD mouse models show that impaired acidification can precede extracellular A*β* plaque deposition, suggesting that it may be an upstream pathogenic event (Lee et al., 2022). Acidification defects are a common consequence of several factors, including neuroinflammatory signaling, metabolic stress (such as mitochondrial dysfunction), lipid toxicity, and the accumulation of toxic protein aggregates (Xiao et al., 2014; Belloy et al., 2020; Zeng et al., 2025). These factors may contribute to impaired v-ATPase function at the lysosomal membrane, resulting in elevated lysosomal pH (Zeng et al., 2025). This, in turn, reduces the activity of acidic hydrolases, such as cathepsins B, D, and L, further compromising the phagocytic and degradative functions of astrocytes (Lo and Zeng, 2023; Zeng et al., 2025). Together, these changes create a self-amplifying pathological vicious cycle.

Overall, AD progression is accompanied by persistent astrocytic lysosomal dysfunction, which in turn impairs A*β* clearance and amplifies inflammation and neurotoxicity (Lo and Zeng, 2023; Wirth et al., 2024; Zeng et al., 2025). Therapeutic strategies should therefore aim to restore lysosomal acidification and degradative flux, while correcting maladaptive A*β* handling and ApoE4-associated defects in lipid trafficking.

### 3.3 Extracellular degradation

The CNS has developed an intricate and efficient proteolytic system to maintain A*β* homeostasis. Astrocytes are integral to this process, functioning as a dynamic and multifunctional center for enzymatic A*β* clearance, effectively mitigating amyloid burden through a range of distinct mechanisms (Son et al., 2016; Radosinska and Radosinska, 2024) (Table 2).

**Table 2:** Comparative overview of major A*β*-degrading proteases (A*β*DPs).

Enzyme	Protease type	Major cellular source	Primary subcellular localization	Major A*β* substrate preference
Neprilysin (NEP) (Yamamoto et al., 2016; Kim et al., 2023)	Zinc Metalloprotease	Neurons (predominantly); also astrocytes, microglia, and vascular cells	Plasma membrane/cell surface	Soluble A*β* (mainly monomeric and low-order oligomeric species)
Insulin-Degrading Enzyme (IDE) (Miners et al., 2009; Son et al., 2016)	Zinc Metalloprotease	Neurons, astrocytes, microglia	Predominantly cytosolic; also endosomal and cell surface/extracellular pools	Soluble A*β* (monomeric, oligomeric)
Endothelin-Converting Enzymes (ECE-1, -2) (Eckman et al., 2001)	Zinc Metalloprotease	Neurons, endothelial cells, astrocytes	Endosomal/acidic intracellular compartments	Soluble A*β*
Matrix Metalloproteinases (MMP-2, -9) (Liu X. et al., 2017; Radosinska and Radosinska, 2024)	Zinc Metalloprotease	Astrocytes, microglia	Secreted/extracellular space	Soluble and fibrillar A*β*

Astrocytes are an important source of matrix metalloproteinases (MMPs), particularly MMP-2 and MMP-9, which contribute to the degradation of multiple A*β* species, including soluble and fibrillar forms (Yin et al., 2006; Radosinska and Radosinska, 2024). Reactive astrocytes in AD animal models, which cluster around amyloid plaques, exhibit increased expression of MMP-2 and MMP-9 (Yin et al., 2006). Pharmacological inhibition of MMPs leads to elevated A*β* levels in the brain interstitial fluid (ISF) and prolongs the elimination half-life of A*β* (Yin et al., 2006). However, MMPs exert dual effects in AD. They may also participate in BBB disruption, cerebral amyloid angiopathy-related injury, neuroinflammation, and synaptic dysfunction (Liu X. et al., 2017; Rempe et al., 2018). Therefore, MMPs should not be viewed as purely protective or detrimental in AD, but rather as context-dependent regulators whose overall impact is determined by disease stage, cellular source, and the balance between amyloid clearance and neurovascular injury (Radosinska and Radosinska, 2024).

In addition to MMPs, NEP and IDE are also major A*β*-degrading enzymes in astrocytes. NEP is primarily localized to the extracellular and synaptic milieu. Its deficiency accelerates A*β* plaque formation more significantly than that of IDE (Yamamoto et al., 2016; Kim et al., 2023). In AD, astrocytes express NEP, and its expression may be further upregulated in plaque-associated reactive astrocytes (Yamamoto et al., 2016; Kim et al., 2023). Recent studies further reveal that Irisin-mediated downregulation of the ERK-STAT3 signaling pathway enhances astrocytic NEP secretion, reducing A*β* burden and improving cognitive deficits (Kim et al., 2023). Astrocytes also support neuronal A*β* clearance by secreting IDE into the extracellular space *via* an autophagy-based unconventional secretion pathway involving RAB8A and GORASP proteins (Son et al., 2016). This pathway is activated by A*β*; however, in mice with impaired autophagy, such as Atg7 hemizygotes, A*β* stimulation leads to reduced IDE levels in cerebrospinal fluid (CSF) (Son et al., 2016).

The protein levels of NEP and IDE may be compensatorily upregulated in early AD, but their enzymatic activity progressively declines with disease progression (Miners et al., 2009; Dorfman et al., 2010). In the case of NEP, neuronal cell model studies have shown that lipid peroxidation products such as 4-hydroxynonenal (4-HNE) can modify NEP, resulting in oxidative inactivation and impaired proteolytic function (de Dios et al., 2019), whereas antioxidant intervention partially prevents this loss of activity (Lim and Han, 2018). These findings suggest that neuroinflammation and oxidative stress impair A*β*-degrading enzyme activity; their alleviation may thus restore enzymatic function and promote A*β* clearance.

## 4 Astrocytic regulation of BBB-mediated A*β* transport

**Figure 2:**
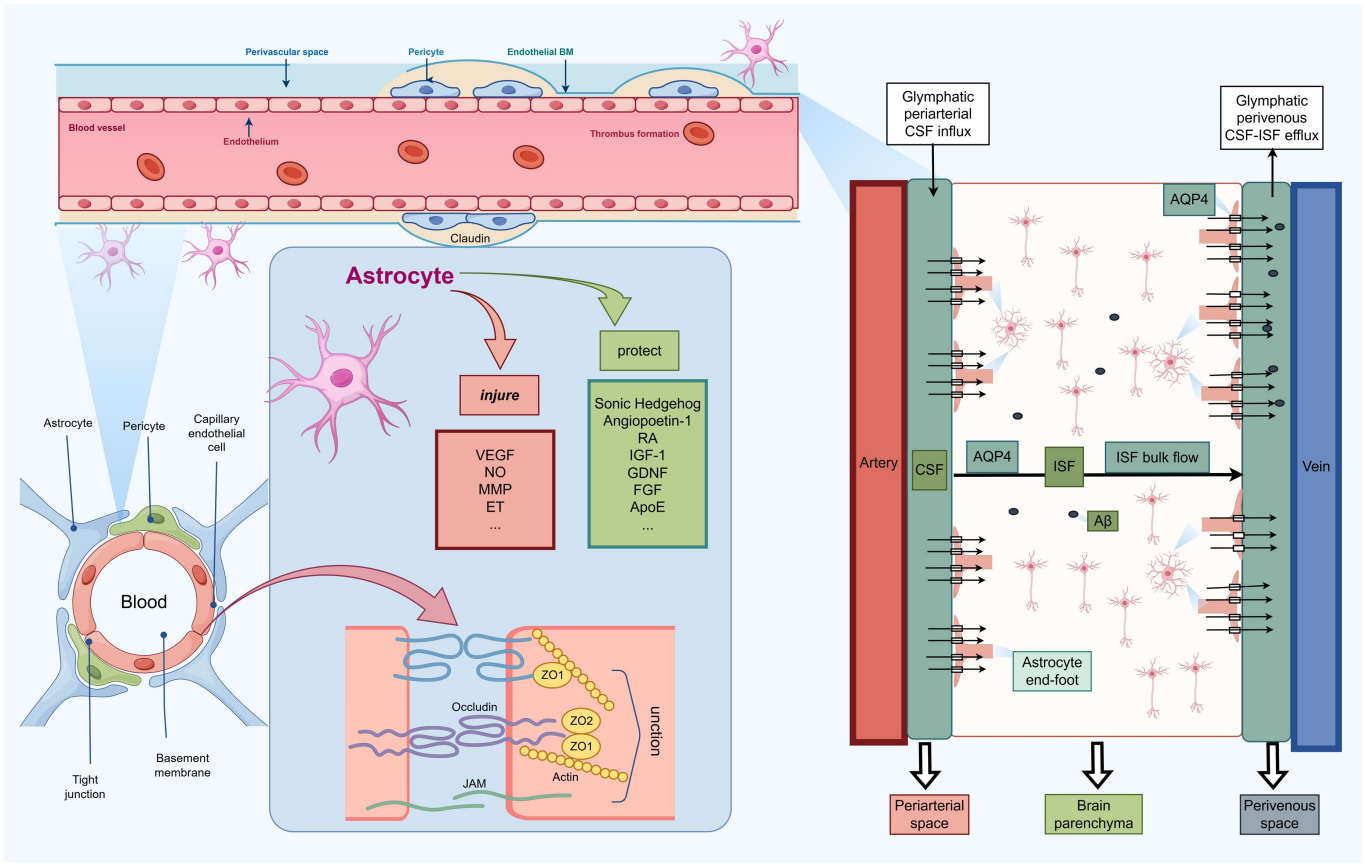
Schematic illustration of astrocyte-mediated regulation of A*β* clearance through the BBB and glymphatic system. The left panel shows the structural organization of the BBB, including endothelial cells, pericytes, basement membrane, tight junctions, and astrocytic endfeet. Astrocytes exert dual effects on BBB function by releasing protective factors, such as sonic hedgehog, angiopoietin-1, RA, IGF-1, GDNF, FGF, and ApoE, or injurious factors, including VEGF, NO, MMPs, and ET, thereby modulating tight-junction integrity and neurovascular stability. The right panel illustrates AQP4-dependent glymphatic transport, in which CSF enters the brain along periarterial spaces, exchanges with ISF through astrocytic endfeet, and promotes perivenous efflux of ISF and A*β*. Under AD-related conditions, reactive astrocytes may impair BBB integrity and disrupt perivascular AQP4 polarization, thereby reducing both BBB-mediated and glymphatic A*β* clearance. The mechanisms and representative mediators shown here are discussed in detail in Sections IV and V, together with the corresponding references. SHH, Sonic hedgehog; Ang-1, angiopoietin-1; TGF-*β*, transforming growth factor-*β*; RA, retinoic acid; GDNF, glial cell line-derived neurotrophic factor; FGF, fibroblast growth factor; IGF-1, insulin-like growth factor-1; ApoE, apolipoprotein E; VEGF, vascular endothelial growth factor; NO, nitric oxide; ET, endothelin.

Compared with microglia and neurons, astrocytes play a more distinctive role in the brain’s trans-barrier clearance system. By regulating the BBB and the glymphatic pathway, astrocytes facilitate the transport of A*β* into the peripheral circulation, where it can be further cleared by peripheral organs such as the liver, kidneys, and intestines (Cheng et al., 2020; Yue and Hoi, 2023). This process directly reduces the cerebral A*β* burden and also minimizes the toxic stress associated with cellular uptake and degradation of A*β* (Cheng et al., 2020) (Figure 2).

### 4.1 Astrocytes in BBB formation and maintenance

The NVU, composed of endothelial cells, pericytes, and astrocytic endfeet, serves as the structural foundation of the BBB (Zhao et al., 2015; Yue and Hoi, 2023). In the pathological progression of AD, the BBB performs a dual role: on one hand, it forms a robust physical barrier with its tight junction architecture, preventing the uncontrolled infiltration of neurotoxic substances—including A*β*, inflammatory mediators, and pathogens—from the peripheral plasma into brain tissue (Zhao et al., 2015); on the other hand, it actively transports A*β* from the brain ISF into circulation for systemic clearance through receptor-mediated transcytosis. Astrocytes are central to both processes.

Astrocytes play a critical role in the formation and maintenance of the BBB. During early brain development, they secrete growth factors such as SHH (Alvarez et al., 2011), Ang-1 (Shen et al., 2011), TGF-*β*, RA (Shearer et al., 2012), and GDNF (Danyang et al., 2022), among others. These factors promote the formation of tight junctions (such as ZO-1 and Occludin) in cerebral microvascular endothelial cells and enhance the expression of transporter systems, thus facilitating BBB maturation (Obermeier et al., 2013; Tietz and Engelhardt, 2015). The astrocytic endfeet also form the “glial limitans,” a critical structure that acts as a second line of defense for the BBB. This structure regulates water homeostasis, ion buffering, and neurovascular coupling, ensuring the stability of the NVU architecture (Hélie-Legoupil et al., 2025). In AD, astrocytes undergo reactive proliferation and shift to a pathogenic secretory profile, transforming into “destructive agents” that release factors such as VEGF (Baumann et al., 2021), NO, MMPs (Liu X. et al., 2017), ROS, and ET (Lin et al., 2014), among others. These factors disrupt the BBB by downregulating and mislocalizing tight junction proteins. Gadolinium-enhanced MRI and histopathological analyses confirm that early BBB damage in AD is characterized by the downregulation of tight junction proteins, reduced perivascular astrocytic coverage, and swelling of terminal endfeet (Nehra et al., 2022). These changes precede the formation of obvious amyloid plaques (Nehra et al., 2022).

### 4.2 Astrocytes in A*β* transport across the BBB

A*β* transport across the BBB is mediated by multiple endothelial receptors, including LRP1, P-glycoprotein (P-gp), and RAGE, whose expression and function are critically regulated by astrocytes (Zhao et al., 2015; Cheng et al., 2020). LRP1 and P-gp facilitate A*β* efflux from the brain into the peripheral circulation (Zhao et al., 2015; Cheng et al., 2020; Li et al., 2025). Astrocytes can modulate LRP1-mediated A*β* clearance in part through the secretion of ApoE, a key molecular chaperone involved in this process (as discussed in Section III.1.3) (Deane et al., 2008). In addition, during the early stages of AD, astrocytes synthesize retinoic acid (RA) via retinaldehyde dehydrogenase-2 (RALDH2), which activates retinoic acid receptors (RARs) in endothelial cells and induces P-gp transcription as a protective feedback mechanism; however, chronic exposure to A*β* eventually suppresses P-gp expression (Shearer et al., 2012). In contrast, RAGE primarily mediates the influx of circulating A*β* from the peripheral blood into the brain. During AD progression, the accumulation of A*β* together with inflammatory mediators released by reactive astrocytes, including TNF-*α*, IL-1*β*, and IL-6, promotes endothelial RAGE upregulation (Donahue et al., 2006; Deane et al., 2012; Li et al., 2025). Meanwhile, the release of MMPs, ROS, and other injurious factors disrupts tight junctions and impairs astrocyte–endothelial interactions. These changes compromise BBB integrity and further aggravate abnormal A*β* transport across the barrier (Liu X. et al., 2017; Yue and Hoi, 2023).

## 5 Glymphatic pathway in A*β* clearance

The glymphatic system is a critical pathway for A*β* clearance, and its optimal function relies heavily on the specialized structures of astrocytes. Recent evidence has demonstrated that enhancing the transport efficiency of this system in AD mouse models significantly reduces A*β* plaque deposition and mitigates neuronal death, highlighting its therapeutic potential in neurodegenerative diseases (Hablitz et al., 2020; Wang et al., 2024).

### 5.1 Structure and function of the glymphatic system

Using two-photon microscopy and magnetic resonance imaging (MRI), researchers have mapped the glymphatic CSF flow patterns in both rodents and humans (Shetty and Zanirati, 2020; Lohela et al., 2022). The glymphatic system is not a set of anatomically distinct ducts but rather a functional perivascular pathway dependent on the concentric architecture of cerebral vasculature and astrocytes. CSF is driven deep into the brain parenchyma through the Virchow-Robin spaces surrounding penetrating arteries, propelled by pressure gradients generated by arterial pulsations and respiratory cycles (Shetty and Zanirati, 2020; Lohela et al., 2022). This influx is mediated by AQP4, the major astrocytic water channel, which is polarized on perivascular and superficial astrocytic endfeet and is essential for glymphatic fluid transport. AQP4 facilitates convective CSF flow along perivascular spaces and across the pial surface into the brain parenchyma (Shetty and Zanirati, 2020; Lohela et al., 2022). Once within the interstitium, this CSF–ISF exchange drives the convective clearance of metabolic waste—including A*β* and tau—toward perivenous drainage pathways (Shetty and Zanirati, 2020; Lohela et al., 2022). Ultimately, this fluid mixture exits the cranium *via* the cribriform plate-olfactory nerve pathway and dural lymphatic vessels, especially basal lymphatics, which handle heavy protein efflux before draining into the deep cervical lymph nodes (Ahn et al., 2019).

### 5.2 Astrocytic AQP4 depolarization and glymphatic failure in AD

During AD progression, disruption of the glymphatic–lymphatic system is driven in large part by the loss of perivascular AQP4 polarization in astrocytic endfeet (Iliff et al., 2012; Kress et al., 2014). Aging, neurodegenerative diseases, sleep disturbances, and abnormal cerebrovascular dynamics further exacerbate clearance failure and are also closely associated with astrocytic AQP4 dysfunction.

Aging and neurodegenerative diseases are associated with loss of perivascular AQP4 polarization. Post-mortem analyses of AD brain tissue consistently reveal a pathological redistribution of AQP4 channels, referred to as “depolarization” (Zeppenfeld et al., 2017), characterized by loss of its normal perivascular localization at astrocytic endfeet and redistribution toward the soma during reactive astrogliosis (Zeppenfeld et al., 2017; Rasmussen et al., 2022). Similarly, in aged mice, loss of perivascular AQP4 polarization is accompanied by impaired glymphatic transport and a marked reduction in A*β* clearance (Kress et al., 2014). This spatial reorganization uncouples AQP4 from paravascular flow pathways, causing ISF stagnation. As a result, sA*β* and tau remain in the parenchyma longer, significantly increasing the likelihood of plaque and NFT formation (Iliff et al., 2012; Kress et al., 2014; Rasmussen et al., 2022). This depolarization is intricately tied to neuroinflammation and reactive astrogliosis. Under normal conditions, AQP4 is tightly anchored to the astrocytic endfoot membrane *via* the dystrophin-associated protein complex (DAPC) (Kress et al., 2014). In inflammatory states, astrocytes upregulate MMP-9, which cleaves the extracellular domain of *β*-dystroglycan, the transmembrane bridge linking the astrocytic cytoskeleton to the basement membrane. This cleavage destabilizes the DAPC and allows unanchored AQP4 to diffuse into the parenchymal membrane. Notably, MMP-9 inhibition has been shown to restore AQP4 polarity and rescue glymphatic function in Parkinson’s disease (PD) models, suggesting that this pathway may represent a potential therapeutic target in AD (Si et al., 2024).

Sleep disturbances compromise glymphatic clearance by modulating ISF flow resistance, but they also directly impair the expression and function of the AQP4. During wakefulness, norepinephrine release from the locus coeruleus constricts the interstitial space, increasing resistance to ISF flow, whereas during NREM slow-wave sleep, ISF resistance is minimized (Reitman et al., 2023). Thus, NREM sleep constitutes a critical window for metabolic waste efflux. PET studies further show that acute sleep deprivation significantly increases A*β* burden in AD-vulnerable regions, particularly the hippocampus and thalamus (Shokri-Kojori et al., 2018). Moreover, growing evidence suggests a direct association between sleep disturbance and AQP4 dysregulation. In humans, chronic insomnia is associated with reduced serum AQP4, Cx30, and Cx43 levels, suggesting astrocytic dysfunction (Yang S. et al., 2022). In mouse models, perivascular AQP4 polarization normally peaks during the rest phase, whereas sustained sleep loss disrupts this pattern and reduces hippocampal AQP4 expression (Hablitz et al., 2020). Subsequent studies found that chronic sleep deprivation upregulates astrocytic AT1R and disrupts AQP4 perivascular localization via the MAPK/Cx43 pathway; inhibition of AT1R restores AQP4 distribution and glymphatic function (Gao et al., 2025).

Abnormal cerebrovascular dynamics involve not only impaired cardiac pumping function but also reduced arterial compliance, the latter being particularly characteristic of chronic hypertension and cerebral small vessel disease. These disturbances primarily diminish glymphatic clearance efficiency by weakening the perivascular driving forces required for fluid transport. For example, animal studies have shown that impaired cardiac function or carotid artery ligation, which leads to abnormal perfusion, can markedly suppress CSF–ISF exchange (Iliff et al., 2013). Similarly, studies in chronic hypertensive mouse models have found that reduced vascular compliance attenuates the transmission of arterial pulsations into the perivascular space, thereby decreasing fluid movement within the PVS (Mestre et al., 2018). In addition, chronic hypertension has been linked to abnormalities in astrocytic AQP4 organization, including altered endfoot-associated expression and disrupted perivascular polarization (González-Marrero et al., 2022). At the population level, an analysis of 1,690 cognitively normal older adults from the A4/LEARN cohort found that elevated baseline pulse pressure was associated with greater global A*β* burden, increased temporal tau deposition, and faster cognitive decline, particularly in APOE ε4 carriers (Jung et al., 2025). Therefore, improving cardiovascular status and cerebral perfusion through regular exercise, together with preserving vascular compliance through appropriate antihypertensive treatment, may represent important strategies to support brain clearance homeostasis, prevent AD, and delay related pathological progression (Jung et al., 2025).

### 5.3 Therapeutic targeting of the glymphatic pathway in AD

The glymphatic pathway is essential for the clearance of cerebral A*β* deposits, and emerging therapeutic strategies focused on this mechanism—encompassing sleep management, exercise and various physiotherapeutic approaches—are reshaping treatment paradigms for neurodegenerative diseases.

Pharmacological Sleep Intervention: While benzodiazepines (BZDs) are commonly used for insomnia, long-term or high cumulative exposure has been associated with an increased risk of dementia, possibly because chronic BZD use suppresses NREM slow-wave activity and thereby impairs glymphatic clearance (Barbaux et al., 2025). Accordingly, in AD, sleep management should prioritize interventions that preserve or restore physiological sleep. In this context, melatonin has been reported to improve glymphatic function, at least in part by restoring perivascular AQP4 polarization, and attenuate reactive astrogliosis in experimental settings (Yao et al., 2023). By contrast, dual orexin receptor antagonists (DORAs) have been reported to prolong sleep duration without markedly disrupting normal sleep architecture, whereas direct evidence for their effects on AQP4 polarization or reactive astrogliosis remains limited (Li et al., 2024). Moreover, Cognitive Behavioral Therapy for Insomnia (CBT-I) has also gained significant clinical attention as an effective non-pharmacological intervention (Bozzelli and Tsai, 2024).

Exercise Intervention: High-Intensity Interval Training (HIIT) improves cardiorespiratory fitness and increases CBF, promoting pulsatile CSF dynamics that enhance metabolic waste clearance. Even in elderly populations with limited tolerance for high-intensity exercise, moderate exercise regimens offer substantial therapeutic benefits (Feng et al., 2023; Liang et al., 2025). Specific activities such as swimming have been shown to enhance AQP4 anchoring by upregulating Lama1 and Dp71 gene transcription, thus preventing AQP4 depolarization (Liang et al., 2025). This exercise-based strategy appears most effective when initiated during the preventative stages of neurodegeneration (Feng et al., 2023).

Physiotherapeutic Interventions: Several non-invasive modalities have shown therapeutic promise. Sun et al. reported that 1 h of 40-Hz multisensory stimulation (light plus sound) significantly increased AQP4 polarization at astrocytic endfeet and markedly accelerated ISF clearance (Murdock et al., 2024). In addition, transcranial photobiomodulation (tPBM) has been shown to improve CBF and tissue oxygenation; it also activates cytochrome c oxidase (CCO) and increases ATP synthesis, suggesting a potential supportive effect on astrocytic energy metabolism (Salehpour et al., 2022; Gaggi and Iosifescu, 2024). McConnell et al. found that FUS induced astrocyte activation with a glioprotective transcriptional signature, while simultaneously promoting meningeal lymphatic vessel (mLV) regeneration and enhancing CSF drainage (Gladwell et al., 2025; Noel et al., 2025).

## 6 Astrocyte-targeted therapeutic strategies for enhancing A*β* clearance

Under pathological conditions in AD, reactive astrogliosis constitutes a central pathogenic event. By impairing A*β* clearance and exacerbating neuroinflammation, reactive astrocytes establish a self-amplifying vicious cycle. Accordingly, current therapeutic strategies should be based on a multi-level interventional framework aimed at interrupting this cycle. As pathway-specific interventions targeting glymphatic dysfunction were discussed in the preceding section, the present section focuses on broader astrocyte-targeted strategies for restoring A*β* clearance and astrocytic homeostasis.

At the level of cellular degradative clearance, therapeutic efforts may be better directed toward enhancing the uptake of the most toxic oA*β*, rather than focusing primarily on poorly degradable plaques (Söllvander et al., 2016). This includes strengthening LRP1–ApoE-mediated clathrin-dependent A*β* uptake. At the same time, intracellular degradative capacity must be restored, including promotion of endosomal–autophagic–lysosomal biogenesis, recovery of lysosomal acidification, and correction of APOE4-associated lipid dyshomeostasis (Lee H. et al., 2023; Lo and Zeng, 2023). In addition, enhancement of extracellular proteolytic degradation may serve as an auxiliary strategy. However, MMPs require careful evaluation, as they may degrade A*β* but also disrupt the BBB and extracellular matrix architecture (Liu X. et al., 2017).

Brain-to-blood A*β* efflux includes both BBB transport and glymphatic clearance, both of which promote the extracranial removal of A*β*, thereby directly lowering the cerebral A*β* burden and reducing the reactive burden placed on local cellular clearance mechanisms (Cheng et al., 2020; Yue and Hoi, 2023). Reactive astrocytes can aggravate neurovascular unit injury and disrupt the polarized distribution of AQP4; therefore, suppressing reactive astrogliosis, modulating neuroinflammatory signaling axes, and reducing the release of destructive factors have emerged as important intervention points for improving A*β* efflux efficiency and the intracerebral microenvironment (Linnerbauer et al., 2020; Toral-Rios et al., 2020; Zeng et al., 2023). The clinical use of anti-A*β* monoclonal antibodies has shown benefits beyond amyloid clearance. For example, donanemab not only reduces aggregated A*β* but also lower plasma GFAP levels, suggesting reduced astrocyte reactivity (Pontecorvo et al., 2022). These findings support combining anti-A*β* immunotherapy with astrocyte-targeted interventions to achieve greater therapeutic efficacy in AD. Finally, a major challenge in AD therapy is limited brain delivery. FUS may address this limitation by transiently opening the BBB, thereby enhancing drug or gene delivery and potentially facilitating A*β* clearance (Gladwell et al., 2025; Noel et al., 2025).

## 7 Discussion and conclusion

Astrocytes are increasingly recognized as central regulators of A*β* homeostasis in AD rather than passive bystanders in disease progression (Linnerbauer et al., 2020; Brandebura et al., 2023). Compared with the more traditionally emphasized role of microglia in plaque phagocytosis, astrocytes exert broader and more integrated control over A*β* clearance by coordinating receptor-mediated uptake, endosomal–lysosomal and autophagic degradation, extracellular proteolysis, maintenance of BBB integrity, and glymphatic transport. Importantly, these functions are highly state-dependent. In the early stages of disease, reactive remodeling of astrocytes may initially represent an adaptive response that helps limit the accumulation of soluble A*β* and preserve neurovascular homeostasis. However, as the disease progresses, astrocytes gradually shift from homeostatic maintainers to amplifiers of clearance failure, neuroinflammation, and neural network dysfunction (Brandebura et al., 2023; Serrano-Pozo et al., 2024). Accordingly, astrocyte-targeted therapies may not only reduce A*β* pathological burden, but also improve BBB integrity, ameliorate the neuroinflammatory milieu, and partially restore neural network homeostasis (Allen and Lyons, 2018; Brandebura et al., 2023).

Nevertheless, several major challenges remain. Current evidence is still derived largely from mouse and cell models, and the translational relevance of many astrocyte-targeted strategies in humans has yet to be established. Astrocyte states in AD display marked heterogeneity across brain regions, disease stages, and genetic backgrounds, making it unlikely that any single intervention will confer uniform benefit in all contexts. Moreover, some of the strategies summarized in Table 3, including TFEB activation, inflammatory reprogramming, senolytic approaches, and gene- or cell-based therapies, may exert pleiotropic effects on multiple CNS cell types beyond astrocytes, which could lead to unintended adverse effects and therefore require further validation (Raha et al., 2021; Yang Z. et al., 2022; Golden et al., 2025). Future studies should therefore prioritize the integration of single-cell and spatial multi-omics, validation in human iPSC-derived and organoid models, and stage-specific *in vivo* investigations to define which astrocyte subpopulations should be targeted, when intervention should be initiated, and how off-target effects can be minimized. Another key challenge is delivery efficiency, as many potentially effective agents remain limited by insufficient brain exposure. This underscores the need for improved vector systems, astrocyte-specific delivery strategies, and enabling platforms such as FUS-assisted BBB opening (Noel et al., 2025).

**Table 3:** Astrocyte-targeted therapeutic strategies for enhancing A*β* clearance in AD.

Category	Representative strategy	Mechanistic rationale	Representative drugs or pathways (astrocyte-related)
A*β* uptake and receptor–chaperone axis	Restoration of surface LRP1 expression	Enhances receptor-mediated uptake and intracellular trafficking of soluble A*β* by astrocytes.	In ApoE4-associated astrocytes, trichostatin A (TSA) and SAHA/vorinostat restore NHE6, correct endosomal hyperacidification, and increase surface LRP1 (Prasad and Rao, 2018b).
	Modulation of ApoE	Enhances ApoE2-mediated A*β* binding and uptake, while ameliorating ApoE4-related endosomal and lysosomal dysfunction.	In APP/PS1 mice, AAV/APOE2 delivery reduces A*β* deposition and improves inflammation (Jackson et al., 2024). In APOE4s2 mice, an inducible allelic-switch model enables efficient conversion from APOE4 to APOE2 after tamoxifen induction (Golden et al., 2025). In APP/PS1 mice, antisense oligonucleotides (ASOs) lower ApoE4 levels (Huynh et al., 2017).
Endolysosomal and autophagy enhancement	TFEB activation	Promotes lysosomal biogenesis and autophagic flux, corrects lysosomal acidification defects, and improves autophagic clearance.	In primary astrocytes, gemfibrozil plus retinoic acid upregulates TFEB via PPARα, thereby enhancing A*β* uptake and degradation (Raha et al., 2021). In APP/PS1 mice, stereotactic intracranial injection of AAV8-GFAP-TFEB lowers ISF A*β* steady-state levels (Xiao et al., 2014). In astrocytes, mTOR inhibition (e.g., rapamycin) promotes TFEB nuclear translocation (Roczniak-Ferguson et al., 2012). In astrocytes, TPK1 upregulation corrects energy metabolic defects and enhances TFEB-associated A*β* clearance pathways (Zhang et al., 2026).
	Correction of lysosomal acidification	Restores hydrolase activity and A*β* degradative capacity by improving lysosomal ion homeostasis and acidification.	In 5xFAD mice, YKL-40/CHI3L1 knockout reduces plaque deposition over a short time course (Zeng et al., 2023). In astrocytes, DEL-C1 reverses CHI3L1-induced cellular dysfunction and restores lysosomal acidification (Kaur et al., 2026). In induced pluripotent stem cell (iPSC), TRPML1 agonists enhance lysosomal activity together with increased acidification (Somogyi et al., 2023).
	Improvement of lipid metabolism	Ameliorates cholesterol trafficking defects that drive lysosomal lipid retention and metabolic dysfunction.	In APOE4-TR mice and human iPSC-derived astrocytes, *β*-cyclodextrin reduces oxysterol/cholesterol burden and lysosomal retention of ABCA1, thereby suppressing mTORC1 activation (Xiao et al., 2014). In APP/PS1 mice, LXR/RXR agonists such as bexarotene increase ABCA1 expression (shown mainly in neuronal models), although dense-core plaque burden does not necessarily decline (Saibro-Girardi et al., 2026).
Extracellular proteases and secretory clearance	Upregulation and secretion of NEP/IDE	Extends A*β* clearance into the extracellular microenvironment through astrocyte-derived A*β*-degrading enzymes.	In primary astrocytes, bilobalide upregulates NEP, IDE, and MMP2, and enhances A*β*42 clearance (Xiang et al., 2021). In astrocytes, irisin promotes NEP secretion and reduces A*β* pathology in 3D AD cultures (Kim et al., 2023).
Inflammatory regulation and reactive signaling	JAK/STAT3	Reprograms astrocytic inflammatory programs, reduces reactive astrogliosis, protects neural networks, and optimizes the microenvironment for A*β* clearance.	In APP/PS1 mice, astrocyte-specific Stat3 knockout attenuates inflammatory factor expression and A*β* plaque pathology (Toral-Rios et al., 2020). In astrocytes, JAK inhibitors [e.g., AG490 (Lee J. Y. et al., 2023), ruxolitinib (Siciliano et al., 2025)] suppress activation of both astrocytes and microglia.
	IL-1α/TNF/C1q axis	Suppresses inflammatory cues that induce the neurotoxic A1-like astrocyte phenotype.	In a spinal cord injury model, anakinra (an IL-1R antagonist) blocks IL-1α-induced astrocyte reactivity (Bretheau et al., 2022). In an LPS-induced neuroinflammation mouse model, etanercept (an anti-TNFα agent) improves behavior and reduces microglial and astrocytic reactivity (Camara et al., 2015).
	P2X7 receptor–NLRP3 axis	Limits inflammasome activation and ATP-driven inflammatory amplification in reactive astrocytes.	In a retinal mechanical injury model, P2X7 receptor antagonists such as BBG, A839977, or A740003 inhibit NLRP3 inflammasome activation (Albalawi et al., 2017).
	NF-κB/IKK2 regulation	Reduces inflammatory transcriptional activation and downstream inflammasome-related signaling.	Natural anti-inflammatory compounds such as curcumin inhibit the NF-κB pathway, downregulate the NLRP3 inflammasome, and reduce inflammatory cytokine secretion (Wang et al., 2025).
Cell therapy and engineered astrocytes	CAR-A engineered astrocytes	Endows astrocytes with the ability to selectively recognize, engulf, and clear pathological proteins, thereby remodeling the local clearance microenvironment around lesions.	CAR-A reduces established or early-forming amyloid pathology in mouse models and induces a potentially resolvable glial response program (Chen et al., 2026).
	Glial progenitor cell transplantation and replacement	Rejuvenates the aged astrocytic niche and may restore AQP4 polarity and supportive functions.	Glial progenitor cell transplantation improves the astrocytic niche in the aged brain and influences AQP4 polarization (Yang Z. et al., 2022).

In conclusion, by delineating the multifaceted roles of astrocytes in A*β* clearance and summarizing recent therapeutic advances, this review not only deepens our understanding of AD pathogenesis but also provides a conceptual framework for the development of earlier diagnostic and therapeutic strategies.
